# Case Report: Neurologic Presentation of West Nile Virus: Difficult Diagnosis

**DOI:** 10.3389/fpubh.2021.628799

**Published:** 2021-12-10

**Authors:** Eron G. Manusov, Amalia Mora Campuzano, Omar Ahmed, Samantha Macias, Carolina Gomez de Ziegler, Gerardo Munoz Monaco

**Affiliations:** ^1^Department of Human Genetics, University of Texas Rio Grande Valley School of Medicine, Mercedes, TX, United States; ^2^Knapp Family Medicine Residency, University of Texas Rio Grande Valley, Mercedes, TX, United States

**Keywords:** West Nile virus, altered mental status, neurologic abnormalities, opsoclonus, pandemic

## Abstract

West Nile virus infections have surged across the globe. South Texas, located on the path of bird migration, with *Culex quinquefasciatus* and other *Culex* species, and biotic primers that predispose the area to epidemics (floods, amplifying hosts, and lack of mosquito control and prevention) remains a highly endemic area for arbovirus spread. West Nile virus infection ranges from mild febrile illness to severe central nervous system involvement. The purpose of this report is to highlight complex presentations of WNV and how confounding presenting symptoms delay diagnosis. The secondary goal is to describe how pandemics, such as SARS-CoV-2, can overwhelm the system and result in medical decision bias errors.

## Introduction

Although first isolated in Uganda in 1937, West Nile virus (WNV, *Flaviviridae*) infection did not establish in the United States as a substantial health concern until 1999 (1–3). In 2002, the WNV infections escalated with over 15,000 laboratory-confirmed cases in 41 American States, Mexico, and Canada. Rapid spread requires biotic primers such as conditions that predispose geographical areas to epidemics, mosquitos, host population dynamics, and meteorological events that increase vector and reservoir populations (4).

Located on the US-Mexico border, South Texas comprises three of the top 20 most impoverished counties in the United States. The prevalence of *Culex quinquefasciatus* and other *Culex* species (primary mosquito vectors for WNV) (5), passerine birds (amplifying hosts for WNV), poor mosquito control prevention, and meteorological events (hurricanes, increased flooding, and rains) create ideal conditions for vector-borne disease. Passerine birds, such as the American robin (*Turdus migratorius)*, American crow (*Corvus brachyrhynchos*), common grackle (*Quiscalus quiscula)*, and house finch (*Haemorhous mexicanus*) are common in the region. Standing ground water is enriched with organic material during storms and is ideal for mosquito larvae growth. The transmission cycle includes passerine birds (reservoir) and *Culex* mosquitoes (vector) with transmission spilling over into horses and humans (dead-end hosts that do not build virus loads sufficient for transmissions) (6). The region is sub-tropical with warmer and wetter seasons that extends the mosquito season from March through October (5).

On the U.S border, we have an extensive surveillance system that identifies vectors, reservoirs, and infected hosts. The pandemic altered the reporting and focus of healthcare providers and the Department of Health and Human Services. The hospitals and emergency departments were filled with patients infected with SARS-CoV-2 and during the scramble, surveillance information was not shared with the clinicians.

West Nile virus infection symptoms can range from mild febrile illness (20%) to severe illness with central nervous system involvement (<1%). Diagnosis is often an after-thought, especially when other viruses (SARS-CoV-2 and Influenza) dominate clinical care. We describe two cases of WNV central nervous system infection diagnosed within 2 weeks in a rural hospital located on the Texas-Mexico border during the 2020 COVID-19 pandemic. During the timeframe, patients infected with SARS-CoV-2 dominated hospital admissions, and inpatient capacity reached a maximum. Although both case reports present with neurologic symptoms on admission, the cases differed significantly. The purpose of this report is to highlight complex presentations of WNV and how confounding presenting symptoms delay diagnosis. The secondary goal is to describe how pandemics, such as COVID-19 can overwhelm the system and result in medical decision bias errors.

## Case Descriptions

This Case Report follows the CARE Checklist of information to include when writing a Case Report. Both patients signed consent for submission.

### Patient 1

A 67-year-old Mexican man presented to the emergency department with weakness, confusion, and lethargy after being discovered obtunded in the back of a semitrailer container where he was living. The patient is a yard worker and was last seen in his usual health 3 days before admission. He was indigent, homeless, without identification, somnolent, minimally responsive, and vaguely denied medication or substance use. He had no known family members, friends, or contacts. The emergency medical services retrieved information from the trailer owner and reported that he would intermittently cut the grass and care for the yard in exchange for a place to rest.

On examination, he was hypertensive (blood pressure=171/36 mm/Hg), afebrile, 17 breaths/min, SpO_2_ = 96% and tachycardia (heart rate = 113 beats/min). Pertinent laboratory results included BUN 43, creatinine 1.4 mg/dL [nl = 0.6.0–1.2 mg/dL], glomerular filtration rate 50.5 [nl>60], aspartate transaminase 138 IU/L [nl = 10–34 IU/L], and alanine transaminase 52 IU/L [nl = 10–40 IU/L] were mildly elevated. The lactic acid was elevated 2.7 mmol/L [nl = 0.5–1 mmol/L], creatine kinase was elevated 4,911 units/liter [nl = 17–150 units/L] (Dimension EXL Integrated Chemistry Siemens). Troponin 0.03 ng/mL [nl = 0–4 ng/mL], urine drug screen positive for opioids, and the urine analysis were positive for 2+ ketones blood, 2+ leukocyte esterase, 2+ protein. His rapid streptococcus test was positive. His chest roentgenography was read as vascular congestion, bronchogram, and cephalization (pulmonary veins of the superior zone dilate due to increased pressure). In the emergency department, he received ceftriaxone 1 g, azithromycin 500 gm, acetaminophen 500 mg, Narcan 4 mg, Clonidine 0.1 mg, and a 2,500 cc Normal Saline bolus.

The patient was admitted to the hospital with the diagnosis of altered mental status and presumptive sepsis, treated with fluid hydration, and ceftriaxone. Doxycycline and Acyclovir were added for possible Typhus and HSV meningitis. Computed Tomography (CT) of the head showed chronic findings without significant findings. SARS-CoV-2 PCR was negative. HIV results, hepatitis panel results, and febrile agglutinins results, were negative. There was no growth from blood/urine cultures.

Our differential diagnosis included substance abuse, urosepsis, meningitis, and endemic typhus. Table 1 outlines laboratory findings. Cerebral Spinal Fluid results from the lumbar puncture, done 3 days after admission day 1, were consistent with aseptic meningitis (pleocytosis elevated protein and predominantly lymphocytic). Immunoassays (enzyme immunoassay-reference range Negative) WNV-specific IgM and IgG were sent to the LabCorp (Burlington 1447, York Court, Burlington, North Carolina) and returned positive within the week. All other PCR and CSF meningitis/encephalitis molecular screening cerebral spinal fluid studies for patient 1 were sent to LabCorp and were negative.

**Table 1:** Laboratory results for patient 1 and patient 2.

**Laboratory**	**Ref range and units**	**Patient 1**	**Patient 2**
Cerebral spinal fluid	Colorless	Colorless	Colorless
WBC	0.00–5/mm^3^	60 mm^3^	29.0 mm^3^
RBC	0.00–5/mm^3^	30 mm^3^	7.0 mm^3^
Cryptococcal antigen	Negative	Negative	Negative
**Meningitis/encephalitis pathogen panel PCR-CSF**			
Enterovirus	Not detected	Not detected	Not detected
*Escherichia coli* K1	Not detected	Not detected	Not detected
Haemophilus influenza	Not detected	Not detected	Not detected
Human herpesvirus 6	Not detected	Not detected	Not detected
Human parechovirus	Not detected	Not detected	Not detected
Herpes simplex virus 1	Not detected	Not detected	Not detected
Herpes simplex virus 2	Not detected	Not detected	Not detected
Listeria moncytogenes	Not detected	Not detected	Not detected
*Streptococcus agalactiae*	Not detected	Not detected	Not detected
*Streptococcus pneumonia*	Not detected	Not detected	Not detected
*Neisseria meningitidis*	Not detected	Not detected	Not detected
Cytomegalovirus	Not detected	Not detected	Not detected
Crypto neoformans/gattii	Not detected	Not detected	Not detected
Varicella-zoster virus	Not detected	Not detected	Not detected
St. Louis Encephalitis	Negative	Positive	Positive
West Nile virus IgG	Negative	Positive	Positive
West Nile virus IgM	Negative	Positive	Positive
WNV confirmatory IgG/IgM	<0.9	N/A	3.61
Fungal Abs, CSF	Negative	Negative	Negative
CMV Abs IgG/IgM	Negative	Negative	Negative
Respiratory pathogen panel-	Not detected	Not detected	Not detected
BioFire respiratory panel 2.1	Not detected	Not detected	Not detected
1. Coronavirus 229E	Not detected	Not detected	Not detected
2. Coronavirus HKU-1	Not detected	Not detected	Not detected
3. Coronavirus NL63	Not detected	Not detected	Not detected
4. SARS CoV by NAA	Not detected	Not detected	Not detected
5. Influenza A PCR	Not detected	Not detected	Not detected
6. Influenza B	Not detected	Not detected	Not detected
7. Parainfluenza virus 1	Not detected	Not detected	Not detected
8. Parainfluenza virus 2	Not detected	Not detected	Not detected
9. Parainfluenza virus 3	Not detected	Not detected	Not detected
10. Parainfluenza virus 4	Not detected	Not detected	Not detected
11. Respiratory syncytial virus	Not detected	Not detected	Not detected
12. *Bordetella parapertussis* by NAA	Not detected	Not detected	Not detected
13. BORDATELLA PERTUSSIS PCR	Not detected	Not detected	Not detected
14. *Chlamydophila pneumoniae*	Not detected	Not detected	Not detected
15. *Mycoplasma pneumoniae*	Not detected	Not detected	Not detected
Varicella-zoster	Negative	Negative	Negative
VDRL	Nonreactive	Nonreactive	Nonreactive
Urine drug screen	Negative	Positive	Not performed
Rapid strep A screen	Negative	Positive	Negative
SARS ag FIA	Negative	Negative	Negative
Past medical history		Homeless and mental status changes. Sepsis	Cardiovascular disease, frequent falls, mental status changes. Sepsis

*The performance characteristic of the Patient 2 WNV assays were validated by: BioAgytix Diagnostics 1320 Soldiers Road Boston, MA 02135. BioAgilytic Diagnostics is a CLIA certified and accredited laboratory for performing high complexity tests. Patient 2 testing was completed at LabCorp Burlington 1447, York Court, Burlington, North Carolina*.

His antibiotics and antiviral medications were discontinued, and the patient was maintained on a high-protein diet and observed for worsening neurologic symptoms (seizures, worsening confusion, gait disturbance). Eventually, his confusion improved. It was impossible to ascertain if he reached his baseline cognitive status without pre-morbid mental status evidence. We could not find a family member, and he had no insurance, and therefore remained in the hospital for 8 weeks until he was discharged to a local homeless shelter.

### Patient 2

One week after the admission of Case 1, a 78-year-old Caucasian male with a significant past medical history of type 2 diabetes mellitus, hyperlipidemia, and coronary artery disease, presented to the same emergency department with a 3-day history of generalized body weakness associated with multiple falls. He described intermittent chills, frontal headache associated with double vision, nausea, and vomiting with no alleviating or aggravating factors.

His blood pressure (BP 127/6 1 mm/hg) and respiratory rate (15 breaths per minute) were normal. His temperature was 100.8°F, Heart rate= 100 beats per minute, white blood count (16 mL, nl = 4 k 11 k/m of blood), and lactic acid (1.78 Nmol/L) were abnormal. No suspicious intra-axial or extra-axial hemorrhage or masses were noted, although there were mild cerebral atrophy and minimal small vessel disease on CT of the head. The C1–C2 relationship with the occipital condyles on CT of the spine appeared unremarkable except for a moderate amount of surrounding degenerative changes. There were also moderate degenerative changes including disc space narrowing at C3–C6 with moderate spinal stenosis extending from C3–C7.

The patient was admitted for presumed sepsis and treated with fluid hydration, and ceftriaxone 1 g daily. Doxycycline was added for possible endemic typhus. On admission, his creatine was 1.6 mg/dl and resolved to 1.2 mg/dl with hydration. He continued to have multiple falls and mental status changes. His SpO2 would drop to 85% with ambulation, his WBC increased to 18 per microliter, and his creatine kinase increased to 365 units/L (Table 1). Three days into the admission, he developed atrial fibrillation with a rapid ventricular response, and he was treated with 5 mg intravenous metoprolol. The differential diagnosis at the time included cord compression, paraneoplastic syndrome, dermatomyositis, Guillan-Barre syndrome, tick-borne associated neuropathy, and Picks Disease. He continued to describe double vision and neurology consult focused on his bilateral lower extremity hyperreflexia. He was evaluated by a neurosurgeon and underwent successful surgical cord decompression. The primary care team ordered a lumbar puncture under-fluoroscopy because of his continued mental status changes and newly developed opsoclonus. The lumbar puncture results were consistent with aseptic meningitis (pleocytosis elevated protein and predominantly lymphocytic). Cerebral spinal fluid studies for patient 2 (listed in Table 1) were positive for the St. Louis Encephalitis and West Nile virus assay. Since there is a significant cross-reactivity, the CSF sample was sent to BioAgilytic Diagnostics and Immunoassays and confirmed WNV-specific IgM =1.4 [reference <0.90] and IgG titers =3.61 [reference <1.30]. The patient remained hospitalized for a total of 3 weeks and was discharged to a rehabilitation center. His mental status and opsoclonus had not resolved at discharge.

## Summary of Cases

One patient was Mexican, and the other Caucasian. During the same period, both patients presented to our hospital with an altered mental status, signs, and symptoms of urosepsis, and diffuse abnormal neurological signs, during the COVID-19 pandemic. Both were treated as per our sepsis protocol and Doxycycline was added because of our endemic typhus prevalence. The social determinants of health varied widely between the two patients, one indigent, without support, finances, and legal immigration status. The other was a retired police officer with a support system, attentive family, and insurance. Both patients tested negative for SARS-CoV-2 and the hospital was at capacity with patients with severe COVID-19 infection. The differential diagnosis for both patients were extensive, and both had mental status changes. Patient 1 underwent an earlier lumbar puncture (3 days after admission) once the clinical course of sepsis resolved. The initial results returned consistent with aseptic meningitis, but the definitive WNV IgG/IgM results returned a week later from a LabCorp laboratory. His clinical course was complicated by hypertension and worsening mental status changes. We were unable to obtain an adequate past medical history without any known patient family or friends. Patient 2 presented with recurrent falls and an initial neurological exam that was focused on signs and symptoms of cord compression. We attributed his falls to diabetic neuropathy and his CT findings to cord compression. The lumbar puncture was ordered immediately after neurosurgery consultation when one of the family medicine residents (AMC) identified the eye findings (opsoclonus) that other physicians did not recognize. The clinical courses for both patients were complicated with confusing neurologic exams, but the lumbar puncture results assisted in making the diagnosis of West Nile virus.

## Discussion

Less than one percent of WNV infections are neuro-invasive; however, those with CNS complications are more likely elderly, patients with alcoholism, and immunocompromised patients (7). Characteristic symptoms of CNS involvement include areflexia or hyporeflexia acute flaccid paralysis without sensory involvement (8). Patients also present with typical meningeal symptoms (neck stiffness, photophobia, headaches, altered mental status, confusion, lethargy, or seizures). Although the long-term neurologic prognosis for most patients with asymptomatic WNV infection is good, upwards of 40% in one study experienced symptoms up to 8 years later (8), and up to 80% of patients who presented with encephalitis continued to have neurologic symptoms long after infection (reduced strength, abnormal reflexes, tremors, impaired immediate and delayed memory) (9). There is a frequent manifestation of movement disorders in patients with neuro-invasive WNV infection, including tremor, parkinsonism, opsoclonus, chorea, athetosis, ballism, and tics.

Patient 1 was found unresponsive, without identification, without support, without finances, or insurance. His mental status was altered to person, place, and time, and the clinical presentation included hypertension, opioids on a urine drug screen, and sepsis. The initial presumptive diagnosis included HSV viral meningitis, bacterial meningitis, and bacteremia. He was started on multiple antibiotics and an antiviral medication that were de-escalated when the clinical picture cleared. He received steroids early. His care was complicated by lack of support, money, home, contacts, or proper identification. He remained in the hospital. After 8 weeks, he could ambulate with a walker, was oriented, but continued with memory loss and inability to perform activities of daily living and instrumental activities of daily living (ADL/IADL).

Patient 2 presented with frequent falls, a history of foot drop, and chronic neurologic deficits from previous lumbar surgeries. An MRI of his cervical spine revealed critical stenosis, and we focused on surgical treatment of the neuro-decompression. Due to COVID-19 visit restrictions, the wife was not at the bedside. After a delay, the primary care team contacted her, and she described her husband's mental status changes. Although his initial neuro-ophthalmologic exam was normal, he developed nystagmus and eventually opsoclonus. Due to concerns about previous lumbar surgery, the lumbar puncture was delayed for 2 days and completed under fluoroscopy in the operating room before the cervical cord decompression surgery.

The diagnosis of West Nile virus is not novel, but the situation (pandemic/geography), regional vector-borne disease prevalence, and psychosocial issues, remind physicians to consider WNV infection in patients with acute and chronic mental status changes. We encountered challenges to diagnosis and management for both patients. First is the focus on COVID-19 infections during the pandemic. The hospital was busy, and resources stretched. We had a sizeable COVID-19 admission rate, and many patients presented with sepsis. Patient 1 presented with acute mental status changes without a source. He was at high risk of a SARS-CoV-2 infection, and we could not quickly obtain a history. He could answer questions during admission but deteriorated quickly.

Although we treated him for sepsis and hypertension, his mental status continued to decline. We completed a lumbar puncture and the results returned consistent with aseptic meningitis, but the WNV results were not available for 5 days. Patient 2 presented with ataxia, falls, and mental status changes in the face of multiple medical problems, prior neuropathy, prior complications of lumbar spine surgery, and sepsis. Initially, we treated his sepsis and chronic illnesses. As an example of anchor bias, we focused on his cervical and lumbar spine findings with his history of lumbar surgery, repeated falls, and increased deep tendon reflexes. He developed opsoclonus, and we changed the focus to the diagnosis to a central nervous system process. A lumbar puncture revealed aseptic meningitis. We entertained WNV due to the endemic arbovirus infections of our region and because of Patient 1. The patient underwent cervical stenosis surgery, was diagnosed with WNV, and was later discharged to a rehabilitation center with significant neurological sequelae.

## Strengths and Limitations of Our Case Report

Arbovirus infections and vectors in South Texas are highly prevalent (10). Even though WNV is rare and neuro-invasive WNV is even more rare, the pandemic affected resources, diagnosis, and critical decision-making. The patient presentations were complex and initially treated with antibiotics, antivirals, and steroids. Care was de-escalated with clinical improvement. Keeping a heightened level of suspicion for arbovirus infection in South Texas is imperative. However, people travel and may end up in areas not used to treating vector-borne diseases, reminding physicians that viral meningitis can make for confusing presentations. The key to both patients was the altered mental status changes. Taking care of critical care issues remains a priority watching for neurological changes and keeping an open mind to the differential diagnosis is crucial. Patient 1's social situation is unfortunate as the severely altered mental status altered the early stages of treatment and long-term placement. We worked with caseworkers, attorneys, legal aid associations, and hospitals to find data and ultimate placement. The team focused on the neurosurgical radiologic findings of patient 2 and ignored the mental status changes, simply attributing them to chronic illness (anchor bias). Once again, the ataxia, personality changes, and movement disorders were critical clues to an accurate diagnosis.

## Patient Perspectives

Due to the COVID-19 pandemic, the emergency department and hospital resources were quickly overwhelmed. Visitor restrictions result in patients staying alone in the hospital. Both patients could not speak to family members, and the varying daily levels of altered mental status made it difficult for the team to obtain accurate histories. Patient 1 eventually improved, and once he was able to provide information, we then focused on rehabilitation and placement. Patient 2 presented with severe cervical spine stenosis and previous lumbar spine surgery. We did not appreciate the degree of altered mental status until we contacted his wife.

## Conclusion

Our purpose is to provide patient cases that can help physicians with critical decision making, battle anchor bias and strengthen the art of creating and adjusting differential diagnoses. These two very different patients were high-risk (homeless, chronic disease, immune-compromised) but presented to us during a pandemic. We learned that it is crucial to exam and re-examine patients and keep a working differential diagnosis that does not anchor us to an original diagnosis. We also learned that our regional arbovirus surveillance systems must include protocols to continue effectively through times of crisis (pandemic).

## Data Availability Statement

The original contributions presented in the study are included in the article/supplementary material, further inquiries can be directed to the corresponding author/s.

## Author Contributions

All authors listed have made a substantial, direct, and intellectual contribution to the work and approved it for publication.

## Conflict of Interest

The authors declare that the research was conducted in the absence of any commercial or financial relationships that could be construed as a potential conflict of interest.

## Publisher's Note

All claims expressed in this article are solely those of the authors and do not necessarily represent those of their affiliated organizations, or those of the publisher, the editors and the reviewers. Any product that may be evaluated in this article, or claim that may be made by its manufacturer, is not guaranteed or endorsed by the publisher.
